# Identification of a Novel Variant of *PDGFC* Associated with Nonsyndromic Cleft Lip and Palate in a Chinese Family

**DOI:** 10.1155/2023/8814046

**Published:** 2023-09-21

**Authors:** Xin Yu, Simin Yang, Wenqian Xia, Xiaorong Zhou, Meiqin Gao, Hui Shi, Yan Zhou

**Affiliations:** ^1^Department of Orthodontics, Prosthodontics and Periodontology, Affiliated Nantong Stomatological Hospital of Nantong University, Nantong, China; ^2^Department of Immunology, School of Medicine, Nantong University, Nantong, China; ^3^Department of Stomatology, Affiliated Maternity and Child Health Care Hospital of Nantong University, Nantong, China

## Abstract

Nonsyndromic cleft lip with or without cleft palate (NSCL/P) accounts for 70% of the total number of patients with cleft lip with or without cleft palate (CL/P) and is the most common type of congenital deformity of the craniomaxillofacial region. In this study, whole exome sequencing (WES) and Sanger sequencing were performed on affected members of a Han Chinese family, and a missense variant in the platelet-derived growth factor C (*PDGFC*) gene (NM_016205: c.G93T: p.Q31H) was identified to be associated with NSCL/P. Bioinformatic studies demonstrated that the amino acid corresponding to this variation is highly conserved in many mammals and leads to a glutamine-to-histidine substitution in an evolutionarily conserved DNA-binding domain. It was found that the expression of *PDGFC* was significantly decreased in the dental pulp stem cells (DPSCs) of NSCL/P cases, compared to the controls, and that the variant (NM_016205: c.G93T) reduced the expression of *PDGFC*. In addition, the Kyoto Encyclopedia of Genes and Genomes (KEGG) pathway analysis showed that *Pdgfc* deficiency disrupted NSCL/P-related signaling pathways such as the MAPK signaling pathway and cell adhesion molecules. In conclusion, our study identified a missense variant (NM_016205: c.G93T) in exon 1 of *PDGFC* potentially associated with susceptibility to NSCL/P.

## 1. Introduction

Cleft lip with or without cleft palate (CL/P) is the most typical congenital developmental deformity in the craniomaxillofacial region, affecting about 1/700 newborns worldwide [1, 2]. Its prevalence rate of newborns in Asia is 1/500 [3, 4], which is higher than that in other regions. Patients with CL/P need to undergo multiple operations to correct the orofacial cleft and solve the accompanying problems, which can cause great psychological, economic, and social burdens [5]. This condition can be divided into syndromic cleft lip with or without cleft palate (SCL/P) and nonsyndromic cleft lip with or without cleft palate (NSCL/P), according to whether it is accompanied by malformations in other organs and systems. NSCL/P patients account for 70% of the total number of patients with CL/P [6] and are currently an area of interest.

The etiology of NSCL/P is complex, which means intrauterine environmental influences affecting synergistically with multiple susceptibility gene variants [7]. Genome-wide association studies (GWAS) and linkage analysis have been conducted to screen multiple NSCL/P genetic risk variants, susceptible genes, and regions such as rs2235371, *NTN1*, 20q12, and 16p13.3[8–10]. Even though these studies have provided a large theoretical basis for the genetic etiology of CL/P, these genes and regions can only explain about 20% of the heritability of NSCL/P [11], and the explanation of heritability is far from expected.

Whole exome sequencing (WES) refers to the targeted capture, sequencing, and analysis of coding regions of the genome using next-generation sequencing (NGS) technologies [12] and is an ideal approach to explore genetic variants associated with clinical phenotypes of patients [13]. It finds common variants, but also low-frequency and rare variants with minimum allele frequencies of less than 5%. To date, many studies based on WES on family inheritance have been reported, and disease-causing variants and genes of some complex genetic diseases have been successfully discovered.

Herein, we conducted WES on NSCL/P patients in a Han Chinese family and screened them for genetic variants according to minor allele frequency (MAF), deleterious prediction, and human genetic disease databases to identify potential pathogenic variants. This study identified a segregating genetic variant in *PDGFC* potentially associated with NSCL/P morbidity, which expanded the genetic pathogenicity profile of NSCL/P in a Han Chinese population and our understanding of its genetic mechanisms.

## 2. Materials and Methods

### 2.1. Human Subjects

Originating from the Central Plain region of China during the early historic [14], the Han Chinese is the largest ethnic group in the world and dominant ethnicity in China [15]. In this research, a Han Chinese family comprising two patients with NSCL/P from Nantong, Jiangsu Province, was recruited at the Nantong Stomatology Hospital. They underwent a comprehensive clinical evaluation by at least two experienced stomatologists to determine the presence of abnormalities, such as congenital heart disease, cyclopia, and polydactyly, to rule out potential syndromes. In addition, we recruited 100 healthy controls of Han Chinese ancestry who were also evaluated and screened by two stomatologists to rule out the absence of NSCL/P. After obtaining the informed consent from the participants or their guardians, we collected 3–5 mL peripheral venous blood of each participant for DNA extraction. The Ethics Committee of Nantong Stomatology Hospital approved this study (PJ 2021-014-01).

### 2.2. Whole Exome Sequencing and Variant Screening

WES was performed on the male proband and his father to generate raw sequencing reads using the NovaSeq 6000 platform. After obtaining the original sequencing data, quality control and data analysis were conducted mapping to the reference genome (GRCh37/hg19). We retained nonsynonymous variants, splicing variants, and frameshift variants. Next, rare variants with MAF ≤ 0.01 were screened using the 1000 Genomes (1000G), Exome Aggregation Consortium (ExAC), and Genome Aggregation Database (gnomAD). In addition, we used the MutationTaster, PolyPhen-2, and SIFT software to assess the harmfulness of the variants. Furthermore, two human genetic disease databases, Online Mendelian Inheritance in Man (OMIM; https://omim.org/) and Human Gene Mutation Database (HGMD; http://hgmd.org/), were used to screen for possible causative genes of NSCL/P.

### 2.3. Sanger Sequencing Validation

Next, we performed the Sanger sequencing on the samples of two patients with NSCL/P (III-1and II-2), asymptomatic mother (II-1) of III-1, and 100 healthy controls. Upstream and downstream gene sequence data were downloaded based on the GRCh37 reference, PCR primers were designed, and the PCR reaction system was configured according to the PCR Master Mix reagent (Illumina, San Diego, CA, United States). The Sanger sequencing data were analyzed using Chromas (Technelysium, South Brisbane, Australia).

### 2.4. *In Silico* Analysis

We assessed the evolutionary conservation of the amino acids corresponding to this variation using the UCSC Genome Browser (https://genome.ucsc.edu/). Platelet-derived growth factor C (*PDGFC*) was modeled from the AlphaFold Protein Structure Database (https://alphafold.ebi.ac.uk/), and the mutant amino acids were analyzed in a three-dimensional structure using the PyMOL software (DeLano Scientific LLC, San Carlos, CA, USA).

Three relevant gene expression datasets from the Gene Expression Omnibus (GEO; https://ncbi.nlm.nih.gov) repository were downloaded for further analysis. GSE42589 contains the gene expression data on dental pulp stem cells (DPSCs) from seven NSCL/P patients and six controls, whereas GSE67985 contains the gene expression profile analysis of craniofacial structures during mouse embryo development from day 10.5 to day 14.5 of the embryo. In addition, by generating *Pdgfc* -/- mutant mice, Andrae et al. explored the developmental role of *PDGFC* in the cerebral cortex [16]. Gene expression changes in cerebral meninges of four *Pdgfc* -/- mutants and nine controls were stored in the GSE67644 dataset.

The clusterProfiler R software package was used to analyze the Kyoto Encyclopedia of Genes and Genomes (KEGG) pathway enrichment of the differentially expressed genes (DEGs) selected in GSE67644. The protein-protein interaction (PPI) networks were constructed using the Search Tool for the Retrieval of Interaction Genes (STRING; https://string-db.org). Next, we searched for hub genes using the cytoHubba plugin in the Cytoscape software.

### 2.5. Cell Culture, Transfection, Real-Time PCR, and Western Blot Analysis

Human embryonic palatal mesenchymal (HEPM) cells and human embryonic kidney 293 (HEK-293), which were cultured in Eagle's Minimum Essential Medium (EMEM), with 10% fetal bovine serum (FBS) and 100 U/mL streptomycin plus 100 U/mL penicillin double antibody, were used for the in vitro experiments.

We cultured HEPM and HEK-293 cells in 12-well culture plates and immediately transfected the DNA plasmid containing either the *PDGFC* G or T allele with Lipofectamine 2000 (Invitrogen, Carlsbad, CA, United States). After 48 hours, we collected the cells, extracted the RNA, and reverse-transcribed it into single-stranded cDNA. The Power SYBR Green on a CFX96 PCR System (Bio-Rad, Hercules, CA, USA) was used to perform real-time quantitative PCR.

Additionally, proteins were extracted at 72 hours following transfection, and their concentrations were detected using a BCA Kit (Beyotime, Songjiang, Shanghai, China). An equivalent amount of protein was isolated on a 12% SDS-PAGE gel and transferred onto PVDF membranes. Next, the membranes were blocked with 5% nonfat milk for two hours and then incubated at 4°C overnight with primary antibodies including anti-PDGFC (Abcam, ab93899) and anti-GAPDH (Beyotime, AG019).

### 2.6. Statistical Analysis

Linear model identification of the DEGs (*P* < 0.05 and |log2(*FC*)| > 1) in GSE67644 was performed by RStudio (R version 4.2.1) based on the GEOquery and Limma R software packages. Volcano plots were generated using the ggplot2 R software package by RStudio. The quantitative real-time PCR results were calculated by a two-tailed Student's *t-*test using IBM SPSS Statistics (version 24), and the significance level was set as *P* < 0.05.

## 3. Results

### 3.1. Clinical Characteristics of the Pedigree with NSCL/P

**Figure 1:**
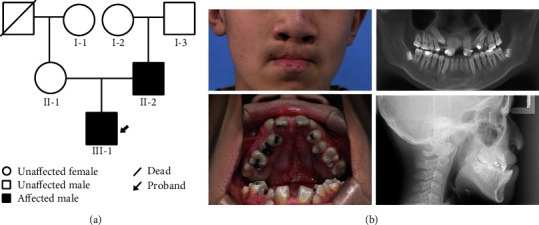
(a) Pedigree structure of the Chinese family with NSCL/P. The proband is marked with an arrow. (b) The facial photograph shows that the proband has a bilateral cleft lip. The panoramic radiograph and intraoral photograph show congenital absence of the maxillary lateral incisors and a cleft palate, while the lateral cephalic radiograph suggests maxillary underdevelopment, presenting a skeletal class III facial pattern.

As shown in the pedigree diagram, we recruited a family of two CL/P patients with paternal relationships for this study (Figure 1(a)). A male proband (III-1) presented with complete CL/P on both sides. The panoramic radiograph showed congenital absence of the maxillary lateral incisors, while the lateral cephalic radiographs suggested maxillary underdevelopment, presenting a skeletal class III facial pattern (Figure 1(b)). In this family, the father of the proband had the same phenotype (II-2), whereas the mother and grandparents were nondysmorphic.

In addition, both patients underwent detailed physical examinations, including eye and vision, outer ear morphology and hearing, cranial development, neuromuscular and cardiovascular system function, and external genital system morphology, to check for the presence of systemic malformations. As a result, we diagnosed both patients with NSCL/P.

### 3.2. A Rare *PDGFC* Variant Identified by WES and Sanger Sequencing

After removing low-quality data, the average clean reads Q20 and Q30 of each sample were 97.2% and 92.5%, respectively, indicating a high sequencing quality. After further quality control, a total of 137,232 unique variants were identified, among which 82,732 variants were shared by the two patients, conforming to the principle of gene-phenotype coseparation.

**Figure 2:**
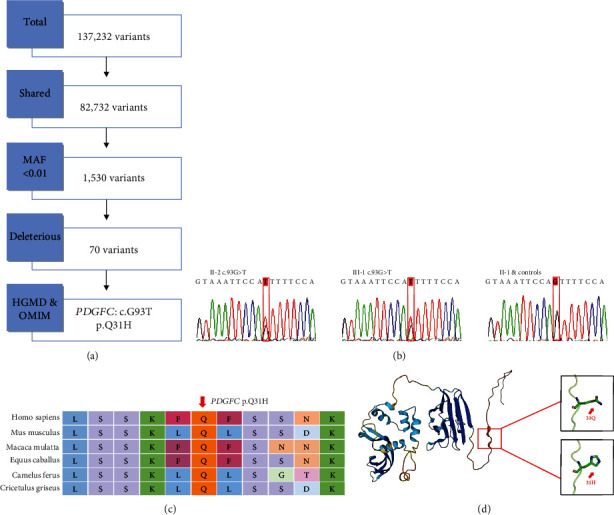
(a) Flowchart of whole exome sequencing filtering strategy. (b) Identification of the mutation in *PDGFC* in two patients with NSCL/P (III-1 and II-2) and asymptomatic mother (II-1) of III-1 by the Sanger sequencing. (c) Comparison of partial amino acid sequences of *PDGFC* proteins in 6 animals. The location of the mutant amino acid is indicated by the red arrow. (d) The predicted three-dimensional model of *PDGFC* with the reference residue (glutamine) and the identified variant (histidine) at position 31.

Subsequently, we filtered and screened the candidate genes after the annotation of variants (Figure 2(a)). Variants located in the exon region were compared with those in 1000G, ExAC, and gnomAD. A total of 1,530 variant sites with MAF < 0.01 were obtained. After removing the synonymous variants, we used several software to predict the effects, which met at least one of the following criteria, including SIFT score ≤ 0.05, PolyPhen-2 HDIV ≥ 0.909, and MutationTaster score ≥ 0.85. Furthermore, two human genetic disease databases, OMIM and HGMD, were used to screen for the possible causative genes of NSCL/P. PDGFC was identified as a candidate-associated gene for CL/P in the HGMD database [17]. A missense variant in *PDGFC* (chr4:157891963, c.G93T: p.Q31H) was identified segregating in this pedigree and further investigated for its association with NSCL/P (Table 1).

In addition, the two patients with NSCL/P (III-1 and II-2), asymptomatic mother (II-1) of III-1, and 100 healthy controls were analyzed using the Sanger sequencing to verify that the variant was pathogenic. A heterozygous variant in *PDGFC* was detected only in the proband and his father (Figure 2(b)), which was consistent with the WES data. Multiple-sequence alignment of *PDGFC* indicated that the amino acids corresponding to this variation were highly conserved in many mammals (Figure 2(c)). In addition, this variant resulted in a glutamine-to-histidine substitution (p.Q31H) in the evolutionarily conserved DNA-binding domain (Figure 2(d)).

### 3.3. *PDGFC* Expression Was Related to NSCL/P and Affected by the Variant

**Figure 3:**
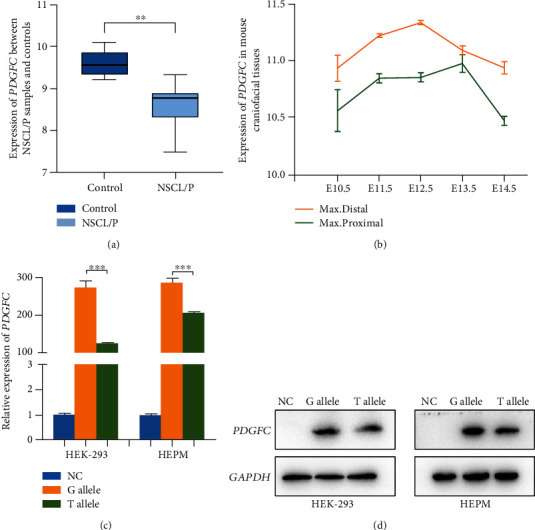
(a) The expression of *PDGFC* in dental pulp stem cells (DPSCs) from 7 NSCL/P cases and 6 healthy controls in GSE42589. (b) The expression of *Pdgfc* in the maxillary structure containing lip and palate tissues during embryonic mouse development (E10.5d-E14.5d). Max. distal: maxillary distal location; max. proximal: maxillary proximal location. (c) *PDGFC* mRNA and (d) protein expression levels in HEK-293 and HEPM cells after transfection of either the *PDGFC* G/T allele or control vector. NC: negative control; ^∗∗^*P* < 0.01; ^∗∗∗^*P* < 0.001.

In order to explore the relationship between the expression of *PDGFC* and NSCL/P, we analyzed the gene chip data in GSE42589, which contained the gene expression data of DPSCs from seven NSCL/P patients and six healthy controls. Compared with the control group, the *PDGFC* expression of DPSCs from NSCL/P patients was significantly reduced (*P* = 0.003, Figure 3(a)). In addition, *Pdgfc* was continuously and dynamically expressed in the mouse maxillary structure containing lip and palate tissues from day 10.5 to day 14.5 of the embryo (Figure 3(b)), suggesting an important role during craniomaxillofacial development.

The effect of this variant (c.G93T: p.Q31H) on *PDGFC* protein and mRNA levels was also studied. We transfected wild-type and mutant expression plasmids of *PDGFC* into HEPM and HEK-293 cells and detected the expression. The variant T allele significantly decreased the expression of *PDGFC* compared to the G allele at both the mRNA (Figure 3(c)) and protein levels (Figure 3(d)). The above evidence suggests that this variant reduces the expression of *PDGFC*, thereby mediating the occurrence and development of NSCL/P.

### 3.4. *Pdgfc* Deficiency Disrupted Signaling Pathways Associated with NSCL/P

**Figure 4:**
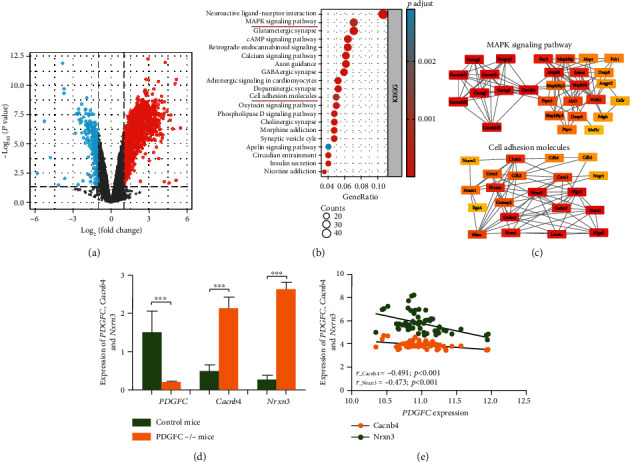
(a) Volcano plots of genes in GSE67644. Red, blue, and gray colors indicate relatively high, low, and equal expressions of genes, respectively. (b) The top 20 pathways in KEGG pathway analyses of differentially expressed genes between *Pdgfc* -/- mutant mice and controls. (c) The PPI network of the MAPK signaling pathway and cell adhesion molecules. (d) The expression changes of *Pdgfc*, *Cacnb4*, and *Nrxn3* in *Pdgfc* -/- mutant mice and control groups. (e) The correlation between *Pdgfc*, *Cacnb4*, and *Nrxn3* expressions in craniofacial structures during embryonic mouse development (E10.5d-E14.5d). ^∗∗∗^*P* < 0.001.

*Pdgfc* -/- mutant mice display a range of severe phenotypes including cerebellar malformation, spina bifida, neuronal overmigration in the cerebral cortex, and palatine insufficiency. DEGs (*P* < 0.05, |log2(*FC*)| > 1) were identified in the cerebral meninges between *Pdgfc* -/- mutant mice and controls (Figure 4(a)). Next, we conducted KEGG pathway enrichment analysis of DEGs to find 47 significant pathways and displayed the top 20 pathways (Figure 4(b)). Among them, the MAPK signaling pathway and cell adhesion molecules are involved in the development of NSCL/P [18–21]. We constructed PPI networks and found that *Cacnb4* and *Nrxn3* were hub genes in the pathways associated with NSCL/P (Figure 4(c). The expression of *Cacnb4* (*P* < 0.001, log2(FC) = 2.19) and *Nrxn3* (*P* < 0.001, log2(FC) = 3.37) was significantly increased in the *Pdgfc* -/- mutant group (Figure 4(d)). In addition, *Pdgfc* expression was significantly and negatively correlated with *Cacnb4* and *Nrxn3* during mouse craniofacial development (Figure 4(e)). As a result, *Pdgfc* deficiency could affect certain key genes, thereby disrupting NSCL/P-related signaling pathways.

## 4. Discussion

Among the 540 CL/P cases studied by Kot and Kruk-Jeromini, 83% were sporadic and 17% were familial [22]. For first-degree relatives of individuals with CL/P, the risk is approximately 32 times higher than the overall risk. There is no significant increase in risk for third-degree relatives compared to the general population [23]. Twin studies also show evidence of genetic etiology, as the concordance rate between monozygotic twins (40%–60%) and dizygotic twins (8%–10%) is consistently high [24, 25]. Although the occurrence of CL/P is related to multiple factors rather than a single etiological mechanism, screening for pathogenic variants in families with CL/P can help us further reveal the genetic susceptibility factors of CL/P and provide a reference for prenatal diagnosis and clinical treatment. In this study, we screened a potential variant of *PDGFC* (c.G93T: p.Q31H) associated with NSCL/P in a Han Chinese family. Through biological function experiments and bioinformatic analysis, the potential mechanism of NSCL/P caused by this variant was explored in depth.

Platelet-derived growth factor C (*PDGFC*) belongs to the platelet-derived growth factor (*PDGF*) family. Several studies have reported that *Pdgfc* knockout mice have a cleft face phenotype, which suggests that *Pdgfc* plays an important role in palatal frame fusion during embryonic growth [26, 27]. In addition, an association between *PDGFC* and CL/P has been demonstrated in population studies. For instance, two neonatal deletions of HSA 4q32 and 4q34 involving *PDGFC* have been reported in a newborn boy with a bilateral cleft of the primary palate and duplicated triphalangeal thumbs [28]. Zhou et al. revealed that rs4691383 and rs7667857 in the *PDGFC* gene were closely related to the occurrence of NSCL/P in the Western Chinese population [29]. Wu et al. also demonstrated an association between rs17035464 on *PDGFC* and NSCL/P in case-parent trios from the Han Chinese population only when considering parent-of-origin effects [30].

*Pdgfc* deficiency disrupts cell adhesion molecules and the MAPK signaling pathway, which were confirmed to be involved in the development of NSCL/P. Craniofacial development is an ordered sequence of events, including structural growth, elevation, attachment, and fusion [31]. Cell adhesion dysfunction can block necessary processes during embryonic development, leading to a cleft [21, 32]. Mammadova et al. also found that the deregulation of oral keratinocyte adhesion can contribute to the pathogenesis of orofacial clefts [33]. The MAPK signaling pathway is one of the important signaling pathways in craniofacial development. It has been reported that MAPK pathway disruption in neural crest cells causes a cleft palate of the Pierre Robin sequence [34]. Furthermore, strict and orderly regulation of MAPK signal is very important in palate development; excessive tamoxifen exposure leads to cleft palate defects in mice by regulating the MAPK signaling pathway [35].

## 5. Conclusion

The study identified a missense variant (NM_016205: c.G93T) in exon 1 of *PDGFC* associated with the susceptibility to NSCL/P. Moreover, based on our experiments, the variant appears to lead to the decreased expression of *PDGFC*, further affecting cell adhesion molecules and the MAPK signaling pathway, resulting in the occurrence of NSCL/P. These findings expand the variant spectrum of *PDGFC* and improve our understanding of its possible association with NSCL/P.

## Figures and Tables

**Table 1:** The information of the variant on *PDGFC* in this NSCL/P family.

Chr	Gene	Mutation site	Mutation type	Minor allele frequency			Deleterious prediction scores			Human genetic disease databases	
				1000G	ExAC	gnomAD	SIFT	Polyphen2_HDIV	MutationTaster	OMIM	HGMD
chr4	*PDGFC*	c.G93T:p.Q31H	Missense	0.001	0.005	0.005	0.216	0	1	—	CL/P

1000G: 1000 Genomes; ExAC: Exome Aggregation Consortium; gnomAD: Genome Aggregation Database; OMIM: Online Mendelian Inheritance in Man; HGMD: Human Gene Mutation Database.

## Data Availability

The gene expression datasets used during the study can be obtained from GSE42589, GSE67985, and GSE67644 in Gene Expression Omnibus (GEO) database. Other data used to support the findings of this study are available from the corresponding authors upon request.
